# A Case of Renal Calculi, with Abscess and Destruction of the Right Kidney

**Published:** 1844-02

**Authors:** R. Martin

**Affiliations:** Nashville, to the Medical Society of Tennessee


Art. III.—*A Case of Renal Calculi, with Abscess and Destruction of the right Kidney.* Reported by R. Martin, M.D., of Nashville, to the Medical Society of Tennessee. May, 1843.

On the 30th of January, 1841, I visited a negro man, the property of W. S. Y., Esq., who, in the latter part of the preceding month, had complained of violent pain in the abdomen, extending along the spermatic cord of the right side. When I saw him he had severe pain between the umbilicus and spine of the ilium, with a prominence of, perhaps, six inches in its several superficial diameters, pretty well defined, and acutely sensible to the touch. Along with an anxious and depressed countenance, a hot, dry skin, small, frequent and slightly tense pulse, his urine was scanty, his tongue nearly natural.

During the month of February his sufferings were materially mitigated by medical treatment, his appetite improved, and he slept pretty well at night; the sensibility of the prominent section of the abdomen abated, and about the first of March the prominence suddenly decreased in size, and was followed, as the patient reported, by the discharge at one time of at least a pint of pus through the urethra, and by smaller quantities at irregular intervals for some days. From that time his apparent improvement was more steady and obvious.

About the first of April, he thought himself able to go out to work, and did plough a part of a day, contrary to my advice, and without his master's permission.

From that time he again declined, the local fulness of the abdomen reappearing, and on the 22d of April, its characteristic local prominence was merged in a less marked, though general increased volume of the abdomen. I did not see him until the 25th; at which time he had distressing and incessant hiccough, shrunken and anxious countenance, feeble, very frequent, thready pulse, violent pain and excessive tenderness over the entire abdomen—position, as when first seen, on the back, with the thighs flexed on the pelvis; skin cool and moist; extremities cold.

May 20th.—Patient died. This morning, corpse greatly emaciated, presenting an unusual dimension, forward and laterally in the upper third of the abdomen, and lower section of the chest—the whole anterior extent of this prominent section sounded dull on percussion. When the sternum was raised a healthy liver was found lying directly under the lower third of that bone, adhering by a very delicate texture of lymph to the anterior concavity of the diaphragm and parietes of the abdomen to its margin. Lower down in the abdomen, the omentum adhered to the walls of the cavity before, and to the intestines behind, and they to each other wherever there was contact. Five pints of consistent inodorous pus were taken from the abdomen, occupying every accessible portion of its cavity, though much the largest collection was found in the posterior part of the right lumbar, extending through the hypochondriac region, encroaching on the chest, by forcing the diaphragm upward, and the liver upward, forward and to the left, until it was brought into the position already described, and also displacing the heart, to some extent. In a word, every thing moveable was carried more or less upward, and to the left.

The peritoneum was here much thickened, and so it was in several other situations. In the chest were some adhesions.

No vestige of the right kidney, in its peculiar renal structure, was detected, but, occupying the right half front of the upper part of the first and second lumbar vertebræ, were found three calculi, enclosed in an enlarged sacculated ureter, the coats of which were very thick.

The bladder contained six or eight ounces of a more fluid pus, and was healthy with two exceptions,—a slight thickening about the orifice of the diseased ureter, and a loss of substance, the superficial extent of which was equal to a shilling piece, and extending nearly through the bladder, while the characteristic features of ordinary ulceration were absent.

Every portion of the large intestines was occupied by a substance, in color an invisible green—wanting the odor of fæces—in consistence ranging from that of cheese to cream. In the rectum it was most firm; the small intestines contained a similar substance in smaller quantity, and of more fluid consistence.

The muscular coat of the intestines pretty generally, but especially of the large ones, was almost black, without any other apparent alteration in their physical structure. The same alteration of color was also observed in the portion of the ureters enclosing the calculi.

The length of time that elapsed from the escape of pus from its original situation, to the death of the patient, is a circumstance worthy of remark.

May, 1843.

